# An Optimized and Simplified System of Mouse Embryonic Stem Cell Cardiac Differentiation for the Assessment of Differentiation Modifiers

**DOI:** 10.1371/journal.pone.0093033

**Published:** 2014-03-25

**Authors:** Matthew E. Hartman, Jason R. Librande, Ivan O. Medvedev, Rabiah N. Ahmad, Farid Moussavi-Harami, Pritha P. Gupta, Wei-Ming Chien, Michael T. Chin

**Affiliations:** Division of Cardiology, Department of Medicine, University of Washington, Seattle, Washington, United States of America; University of Milan, Italy

## Abstract

Generating cardiomyocytes from embryonic stem cells is an important technique for understanding cardiovascular development, the origins of cardiovascular diseases and also for providing potential reagents for cardiac repair. Numerous methods have been published but often are technically challenging, complex, and are not easily adapted to assessment of specific gene contributions to cardiac myocyte differentiation. Here we report the development of an optimized protocol to induce the differentiation of mouse embryonic stem cells to cardiac myocytes that is simplified and easily adapted for genetic studies. Specifically, we made four critical findings that distinguish our protocol: 1) mouse embryonic stem cells cultured in media containing CHIR99021 and PD0325901 to maintain pluripotency will efficiently form embryoid bodies containing precardiac mesoderm when cultured in these factors at a reduced dosage, 2) low serum conditions promote cardiomyocyte differentiation and can be used in place of commercially prepared StemPro nutrient supplement, 3) the Wnt inhibitor Dkk-1 is dispensable for efficient cardiac differentiation and 4) tracking differentiation efficiency may be done with surface expression of PDGFRα alone. In addition, cardiac mesodermal precursors generated by this system can undergo lentiviral infection to manipulate the expression of specific target molecules to assess effects on cardiac myocyte differentiation and maturation. Using this approach, we assessed the effects of CHF1/Hey2 on cardiac myocyte differentiation, using both gain and loss of function. Overexpression of CHF1/Hey2 at the cardiac mesoderm stage had no apparent effect on cardiac differentiation, while knockdown of CHF1/Hey2 resulted in increased expression of atrial natriuretic factor and connexin 43, suggesting an alteration in the phenotype of the cardiomyocytes. In summary we have generated a detailed and simplified protocol for generating cardiomyocytes from mES cells that is optimized for investigating factors that affect cardiac differentiation.

## Introduction

In vitro systems to differentiate pluripotent stem cells to cardiac myocytes have been invaluable in determining the mechanisms that regulate cardiac differentiation and subtype specification into nodal, working, and conduction system myocardium. Although multiple protocols exist, often they are technically challenging, complicated and give variable yields, which may limit wide adoption. The development of a well-defined, simplified differentiation protocol that is easily adapted for genetic studies will likely make this area of investigation more accessible.

Initially, cardiac differentiation of mouse embryonic stem (mES) cells used the formation of three dimensional, solid spheres of embryonic stem (ES) cells in suspension, known as embryoid bodies (EBs) followed by stimulation with high amounts of serum [1]. This method generally results in a yield of approximately 1–5% cardiomyocytes out of the total cells (reviewed in Boheler et al., 2002) [2]. Kattman et al. have developed a method of directed differentiation of mES cells into cardiomyocytes using timed stimulation with the nodal analog, activin A, and bone morphogenetic protein 4 (BMP4) [3], [4]. This system has the advantage of using cell surface proteins to track the efficiency of cardiac differentiation and reportedly results in 60–80% yield of cardiomyocytes. However following the formation of cardiac mesoderm as evidenced by Nkx2–5, Flk-1, and platelet derived growth factor α (PDGFRα) expression, there can be significant inter-experiment variability in terms of cardiomyocyte yield. This variability potentially limits the utility of these protocols in assessing effects of exogenous genes.

One other common technical hurdle with ES cell culture is the tendency for cultured cells to differentiate and lose their pluripotency, even in the presence of leukemia inhibitory factor (LIF). To address this issue, others have pioneered the use of small molecule inhibitors that target specific signaling pathways to maintain self-renewal and pluripotency. Inhibition of MAPK/ERK kinase (MEK) promotes pluripotency by blocking differentiation signals autoinduced by FGF-4 in cultured mES cells [5]. Blocking glycogen synthase kinase 3β (GSK3β) improves the viability of mES cells cultured in serum free conditions [5]. CHIR99021 and PD0325901 are very specific inhibitors of GSK3β and MEK respectively [6]. Combining these two inhibitors together with LIF in mES cell culture, termed ‘2i+LIF’, results in homogeneous expression of pluripotency markers such as Nanog, Oct4, and Rex1 as well as ability to derive ES cells from various mouse strains [7], [8], including recalcitrant strains like NOD mice [9]. Importantly 2i has been used to derive ES cells from rats [10], [11] and generate naïve porcine induced pluripotent stem cells [12]. One caveat associated with the use of these inhibitors, however, is that their removal must be carefully orchestrated to promote differentiation and viability of differentiated cells, as has been demonstrated in rat ES cells [13].

Here we describe a reliable and efficient method for the differentiation of 2i+LIF cultured mES cells into cardiomyocytes. This system is well-suited for evaluating either positive or negative effects of potential cardiac differentiation modifiers using lentiviral mediated gain or loss of function initiated at the cardiac precursor stage, because the yield of cardiomyocytes is in the intermediate range and the use of viral transduction after differentiation has been initiated provides an internal control for differentiation efficiency. We demonstrate the efficacy of this system in testing the effect of CHF1/Hey2, a bHLH transcription factor expressed in the developing ventricular myocardium, on the cardiac differentiation of mES cells.

## Methods

### Cell Culture and Passage

E14.Bry-GFP (provided by Gordon Keller) [14] and E14.RP11–88L12.Nkx2–5-EmGFP (purchased from MMRRC, www.mmrrc.org) [15] mouse embryonic stem (mES) cells were grown at 37°C with 5% CO_2_ and 21% O_2_ in adherent culture on 0.1% gelatin coated tissue culture plates in ES-20%: Dulbecco’s modified eagles medium (DMEM) supplemented with 20% heat-inactivated embryonic stem cell grade fetal calf serum (FCS), 100 units/ml penicillin, 100 μg/ml streptomycin. Prior to use ES-20% was supplemented with 1.5×10^−4^ M monothioglycerol (MTG) and 1000 units/ml Leukemia inhibitory factor (LIF, Millipore); this working media was kept at 4°C and used for up to 2 weeks. Immediately prior to use in cell culture, 2i (PD0325901 and CHIR99021, Stemgent) was added to final concentration of 0.4 μM PD0325901 and 3 μM CHIR99021. Media was changed at a minimum of every 2 days.

Cells were passaged for continued cell culture or use in differentiation experiments between 3 and 7 days after initial plating as follows. Cells were rinsed gently once with room temperature sterile phosphate buffered saline (PBS). Then we added 1 ml of 37°C Trypsin-like enzyme (TrypLE, Invitrogen) per well of a 6-well plate and incubated at 37°C for 4 min. Cell dissociation was stopped with addition of 4°C sterile 10% FCS-PBS and gently lifting cells from the plate using a P1000 pipetman. Cells were collected and washed twice with room temperature sterile PBS, all with collection at 400 g for 5 min. Cells were then resuspended in ES-20% with 2i for cell culture and plated on 0.1% gelatin coated tissue culture plates or resuspended in serum free conditions for differentiation.

HEK293T cells were grown in adherent culture on tissue culture flasks in 10% FCS DMEM with 100 units/ml penicillin and 100 μg/ml streptomycin at 37°C in 5% CO_2_. Cells were passaged by washing once with media followed by treatment with 37°C 0.05% trypsin-EDTA for 3 min. Cells were then washed and replated at the desired concentration.

### Cardiomyocyte Directed Differentiation

#### Day 0, Formation of EBs

Cells were collected as above and resuspended at 1×10^5^ cells/ml in serum free differentiation media (SFDM, Invitrogen) with or without 2i as designated. In general, for days 0 to 2 EBs were formed in the presence of 0.1 μM PD0325901 and 0.75 μM CHIR99021 (0.25× of the 2i maintenance concentrations). SFDM was prepared as 750 ml Iscove’s modified Dulbecco’s medium (IMDM, Invitrogen) supplemented with 250 ml F12 (Invitrogen), 5 ml 100X N2 (Invitrogen), 10 ml 50X B27 without retinoic acid (Invitrogen), 0.05% bovine serum albumin (BSA), 100 units/ml penicillin, and 100 μg/ml streptomycin and stored as frozen aliquots. Working solution was prepared by adding 4.5×10^−4^ M MTG and stored at 4°C for up to 2 weeks. Immediately prior to use with cells we added 2i at the designated concentration and ascorbic acid to a final concentration of 0.5 μM. To form EBs, 10 ml of cell suspension was transferred to a 10 cm Petri dish and maintained at 37°C in 5% CO_2_ and 21% O_2_. This was designated Day 0, Time 0 of the differentiation protocol.

#### Day 3, Removal of 2i

Approximately 72 (±3) hours after the start of the protocol, EBs were collected by washing the Petri dish once with 5 ml of room temperature sterile PBS while maintaining the Petri dish at a 20°to 30°angle and transferring the 15 ml of cell suspension to a sterile conical tube. EBs were pelleted at 123 g for 3 min, gently washed once with room temperature sterile PBS, repelleted, and then gently resuspended in 10 ml of SFDM without 2i and transferred to a new 10 cm Petri dish. Gentle washing and replating was performed to minimize damage to the EBs.

#### Day 4, Cardiac Mesoderm Formation

A 20X solution of human vascular endothelial growth factor (VEGF), human Activin A (Activin A), and human bone morphogenetic protein 4 (BMP4) was prepared in 37°C SFDM with 4.5×10^−4^ M MTG and 0.5 μM Ascorbic acid. This solution of factors was added at 0.5 ml to each Petri dish 24 (±1) hours after removal of 2i to yield a 1∶20 working dilution; approximately 0.3 to 0.4 ml of media evaporated during incubation from day 3 to 4. Optimal working concentrations were determined by testing each lot of Activin A and BMP4 (table 1). For the experiments shown in this paper the concentrations used are as follows except where noted: 5 ng/ml VEGF, 4 ng/ml Activin A, 10 ng/ml BMP4.

**Table 1:** List of genes and corresponding primers used for qRT-PCR with relative cardiac subtype expression denoted.

Gene	Primers 5′ to 3′	Subtype
Alpha myosin heavy chain (αMHC)	(f) GCCCAGTACCTCCGAAAGTC (r) GCCTTAACATACTCCTCCTTGTC	A>V*
Atrial natriuretic factor (ANF)	(f) GGGGGTAAGGATTGACAGGAT (r) ACACACCACAAGGGCTTTAGG	A ≥ V*
Beta myosin heavy chain (βMHC)	(f) GCCCAGTACCTCCGAAAGTC (r) GCCTTAACATACTCCTCCTTGTC	V>A*
L-type Ca^2+^ channel, Cav1.3 (CACNA1D)	(f) CCCAAAGAAAACGTCAGCAATAC (r) CGGCTGTTTAGACGAGTTACCC	N>A>V
CHF1/Hey2	(f) TGGGGAGCGAGAACAATTAC (r) TTTTCAAAAGCTGTTGGCCACT	V
Connexin 40 (Cx40)	(f) GGTCCACAAGCACTCCACAG (r) CTGAATGGTATCGCACCGGAA	A>V
Connexin 43 (Cx43)	(f) ACAGCGGTTGAGTCAGCTTG (r) GAGAGATGGGGAAGGACTTGT	V>A
Hyperpolarization-activated and cyclic-nucleotidegated channel 4 (HCN4)	(f) ACCCGCAGAGGATCAAGATGA (r) TGCGAGTCTCCACTATAAGGAA	N>A ∼ V
Transient outward K^+^ channel, fast; Kv4.2 (KCND2)	(f) TCAGGACGCTCTGATAGTGCT (r) TCTGGGTATCGTTCCAGGGTG	N>A ∼ V
Inward rectifier K^+^ channel, Kir2.1 (KCNJ2)	(f) ATGGGCAGTGTGAGAACCAAC (r) CTCCGAAGAGACGATGCTGTA	V>A>N
mS16	(f) AGGAGCGATTTGCTGGTGTGGA (r) GCTACCAGGCCTTTGAGATTGGA	N.S.
Myosin light chain-1a (mlc-1a)	(f) AAGAAACCCGAGCCTAAGAAGG (r) TGGGTCAAAGGCAGAGTCCT	A ≥ V*
Myosin light chain-1v (mlc-1v)	(f) ACGCTGGGTGAGAGACTGA (r) CAGCCGTTGGAGTCCTCTT	V ≥ A*
Myosin light chain-2a (mlc-2a)	(f) CTCTTCCTTGTTCACCACCC (r) TCTTTCCTCACACTCTTCGGG	A ≥ V*
Myosin light chain-2v (mlc-2v)	(f) AGGACGAGTGAACGTGAAAAAT (r) GCCCCTTTAAGTTTCTCCCCAA	V>A
Troponin t2, cardiac (Tnnt2)	(f) CAACATGATGCACTTTGGAGGGT (r) TCGCAGAACGTTGATTTCGTATT	N.S.

(f): forward primer, (r): reverse primer. Cardiomyocyte subtypes are A: atrial, V: ventricular, N: nodal. *: Expression depends on maturation, N.S.: not specific. [40]–[43].

#### Day 5+4 hours, Plating of Cardiac Progenitors

EBs were collected as above 28 (±0.5) hours after addition of Activin A and BMP4 with 5 ml room temperature sterile PBS and pelleted at 123 g for 3 min. EBs were dissociated by incubating in 1 ml of 37°C TrypLE for 2 min, gently resuspending three times with a P1000 pipetman, incubating for another 2 min at 37°C, gently resuspending three times with a P1000 pipetman, then adding an equal volume of 4°C 10% FCS-PBS and mixing twice gently followed by pelleting at 4000 g for 5 min. Cells were washed twice with 1 ml room temperature sterile PBS, pelleted as above between washings, counted on the second wash with a hemocytometer, resuspended to 1×10^6^ cells/ml in StemPro media, and then plated at 3×10^5^ cells/well in 0.1% gelatin coated 96-well tissue culture plates. StemPro media used at this stage was supplemented with 100 units/ml penicillin, 100 μg/ml streptomycin, 4.5×10^−4^ M MTG, 1% FCS, VEGF 5 ng/ml, 2 mM L-glutamine and kept at 4°C for up to 1 week. Immediately prior to use ascorbic acid was added to a final concentration of 1 μM. StemPro media was changed every 2 days starting on day 7 with 0.2 ml of new StemPro media per well of a 96-well plate.

### Extracellular and Intracellular Protein Staining and Flow Cytometry

For surface protein staining and evaluation of green fluorescent protein (GFP) expression, EBs on day 5 of the differentiation protocol were dissociated, washed as above, resuspended in 100 μl 4°C 5% FCS-PBS, and incubated on ice for 15 min in the dark with 0.2 μg/ml of both Cy5.5-labeled anti-Flk1 and phycoerythrin (PE)-labeled anti-PDGFRα. Following incubation, an equal volume of 10% FCS-PBS was added and cells were kept on ice and in the dark until analyzed by flow cytometry.

For intracellular staining of cardiac troponin (Tnnt2), mES cells were washed once with room temperature sterile PBS and dissociated with 100 μl/well of 37°C 0.25% trypsin-EDTA containing 100 units/ml DNAse I for 5 min followed by addition of 100 μl/well of 37°C 0.25% trypsin-EDTA with 100 units/ml DNAse I. Cells were kept at 37°C and monitored every 5 min for a morphologic change to a round appearance with separation of neighboring attachments. Once this was observed, cells were mixed with a P200 three times and the reaction was stopped by addition of an equal volume of 50% FCS-PBS with 100 units/ml DNAse I. Cells were collected at 400 g for 5 min, fixed in 100 μl of 4% paraformaldehyde at room temperature in the dark for 10 min. Cells were collected at 2000 g for 5 min (used for all subsequent collections). Cells were washed in 5% normal goat serum (NGS)-PBS for 10 min at room temperature in the dark, collected, resuspended in 100 μl 4°C 0.75% saponin-5% NGS-PBS with 2 μg/ml anti-Tnnt2, and incubated for 30 min at room temperature in the dark. Cells were washed twice with 4°C 0.75% Saponin-5% NGS-PBS, resuspended in 100 μl 4°C 0.75% saponin-5% NGS-PBS with 20 μg/ml goat anti-mouse IgG-AlexaFluor546, and incubated for 30 min at room temperature in the dark. Cells were washed once with 4°C 0.75% saponin-5% NGS-PBS, then once with 4°C 5% NGS-PBS, then resuspended in 300 μl 4°C 5% NGS-PBS and kept on ice in the dark until analyzed by flow cytometry. A Becton Dickinson ARIA II flow cytometer was used to collect data and Flowjo version 10 was used for analysis.

### Lentivirus Production and Titer

Using established methods we cloned a CHF1/Hey2 cDNA into the lentiviral expression construct iDuet101a, which contains a tet operator, an EF-1 promoter and a hygromycin resistance cassette [16]. This system has been used for inducible expression in human embryonic stem cells, expresses constitutively in the absence of the tetracycline-responsive suppressor protein, and is also functional in mouse ES cells. We purchased pLKO.1 plasmids, which have a U6 promoter, containing scramble small hairpin RNA (shRNA) (sequence: 5′ CCT AAG GTT AAG TCG CCC TCG CTC GAG CGA GGG CGA CTT AAC CTT AGC 3′) or CHF1/Hey2 shRNA (sense sequence 5′ CCA CCT CTC TTC TGT CTC TTT3′) from OpenBiosystems (Division of Thermo Scientific). We generated virus by transfection of HEK 293T cells with this plasmid in conjunction with the helper plasmids pMD2.G and psPAX2. Cell culture supernatants containing virus were filtered through 0.45 μm filters, concentrated by ultracentrifugation using PEG-it (Systems Biosciences, Mountain View, CA) and titered by HIV-1 p24 ELISA (Advanced Biosciences Laboratories, Kensington, MD). ES cells were transduced on day 5 of the differentiation protocol at MOI 10 without subsequent selection. Control cells were transduced with a lentivirus containing the iDuet101a plasmid lacking an insert or with the pLKO.1 plasmid with scramble shRNA.

### RNA Isolation and Quantitative RT-PCR

Total RNA was isolated from an entire well of cells using an RNeasy kit (Qiagen) or a GeneJET RNA purification kit (Thermo Scientific) according to the manufacturer’s instructions. RNA was subsequently reverse transcribed into cDNA using iScript cDNA Synthesis kit (BioRad) according to the manufacturer’s instructions. Quantitative real-time PCR was performed on cDNA samples using iTaq Universal SYBR Green Supermix (BioRad) and an Applied Biosystems 7500 Fast Real-Time PCR System (Invitrogen) according to standard protocols and the manufacturer’s instructions. In all experiments the mS16 gene was used as a control. The primers used for the real-time PCR are listed in table 1.

Data analyses were performed with the −ΔΔ C_T_ method, where C_T_ is cycle threshold, as previously described [17]. In brief, −ΔΔ C_T_ is the change in the difference of C_T_ values between the gene of interest and the reference gene over two time points or treatments. At maximal RT-PCR efficiency and equal PCR amplification rates between genes a −ΔΔ C_T_ value of 1 would equate to a doubling of gene expression. However without knowing the PCR efficiency one can use the −ΔΔ C_T_ values as a measure of change in the gene of interest.

### Statistical Analysis

All data are reported as mean ± SEM. Comparisons between groups were made using an unpaired Student’s *t*-test (two groups). Correlation analysis was performed using least squares fit simple linear regression. All analyses were performed using commercially available software (Excel, Microsoft, Redmond, WA). *P* values of <0.05 were taken as the minimal level of significance.

### Cell Imaging

Photographs of embryonic stem cells and embryoid bodies were taken on a Zeiss Axiovert 200 inverted microscope. All settings including exposure time were set to be the same for each picture taken. A picture of a micron ruler was also taken at the same magnification as the embryoid bodies and was used to digitally insert a scale bar using ImageJ version 1.47.

## Results

### Stepwise Removal of 2i Improves the Viability of Embryoid Bodies

**Figure 1:**
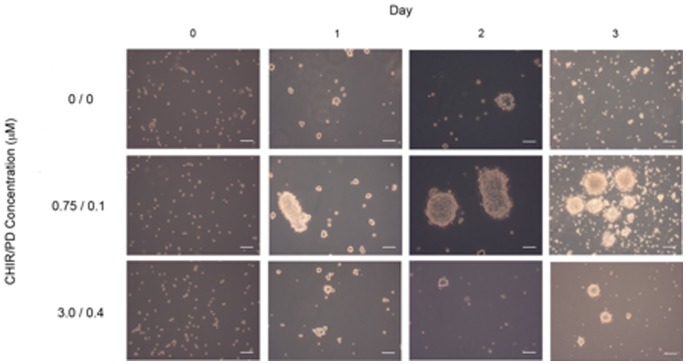
Stepwise removal of 2i improves the viability of embryoid bodies. mES cells cultured in the presence of 2i (3 μM CHIR99021 and 0.4 μM PD0325901) cells were dissociated and transferred to suspension culture to form EBs either in the absence of 2i (0 μM/0 μM), 25% of the original concentration of 2i (0.75 μM/0.1 μM), or “full” 2i (3 μM/0.4 μM). Pictures were taken at the time of plating (day 0) and on days 1, 2, and 3. The scale bar represents a length of 100 microns.

Removing LIF, the presence of which helps maintain pluripotency, is a necessary step in the differentiation of mouse embryonic stem cells into the three germ layers [18]. Since the 2i formulation prevents the expression of the mesodermal marker brachyury [5], these inhibitors must also be removed from culture prior to proceeding with cardiac differentiation [19]. However it is not clear how best to do this with mouse embryonic stem cells cultured in the presence of 2i. Cao et al. developed a method for differentiating 2i cultured rat embryonic stem cells into cardiomyocytes, which uses a stepwise reduction in the amount of 2i to produce viable EBs [13]. We therefore tested the stepwise removal of 2i in the beginning stages of our differentiation protocol. We dissociated mES cells and transferred them to a suspension culture without LIF in order to form EBs, in the presence of 0/0 μM, 0.75/0.1 μM, and 3/0.4 μM of CHIR99021/PD0325901 (fig. 1). We determined that mES cells are unable to form EBs efficiently with the abrupt removal of 2i immediately following the transition from adherent culture to suspension culture. However, with reduced (0.75/0.1 μM) and maintenance culture (3/0.4 μM) concentrations of 2i, mES cells readily formed large EBs within 24 to 48 hours. Cao et al. demonstrated that 0.75/0.1 μM of CHIR99021/PD0325901 was the minimum requirement for forming well-shaped EBs and this concentration of 2i showed a peak cardiac differentiation effect in conjunction with a Rho-associated kinase inhibitor [13]. Therefore, we selected reduced 2i as the concentration for EB formation.

**Figure 2:**
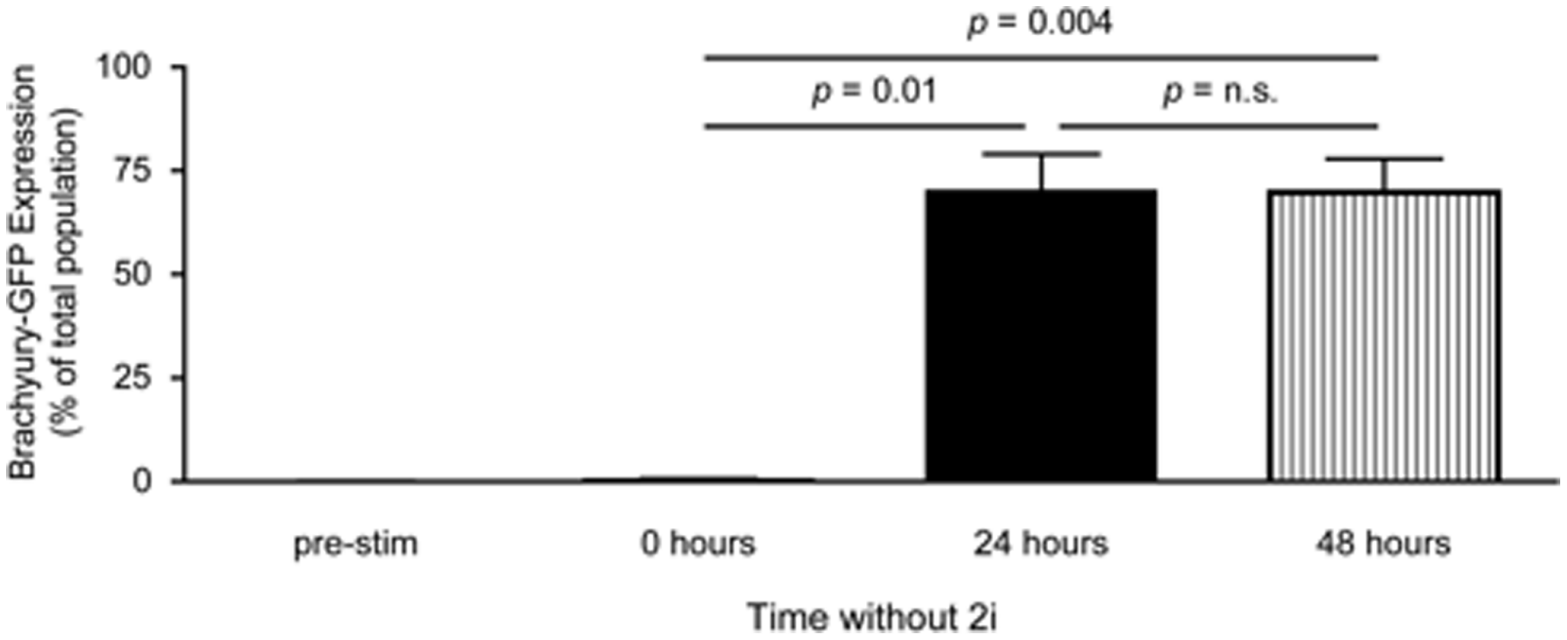
Removal of 2i is required for brachyury expression. EBs were formed from E14.Bry-GFP mES cells in the presence of 0.75 μM CHIR99021 and 0.1 μM PD0325901 or 0.3 μM CHIR99021 and 0.04 μM PD0325901. Either 48 or 72 hours after EB formation, cells were collected and washed once to remove 2i. A subset of cells (pre-stim) was dissociated with TrypLE and brachyury-GFP fluorescence was assessed using flow cytometry (n = 7). The remainder of the EBs were resuspended in SFDM without 2i and treated with 5 ng/ml VEGF, 8 ng/ml Activin A and 1 ng/ml BMP4 either immediately (0 hours, n = 2), 24 hours (n = 3), or 48 hours (n = 5) after 2i removal. After 26 to 29 hours of treatment, EBs were dissociated with TrypLE and brachyury-GFP fluorescence was assessed using flow cytometry.

We further examined the effect of reduced 2i on the differentiation of EBs using a mES cell line which expresses GFP driven by the brachyury promoter to help assess the formation of mesoderm [14]. We observed that we had to remove the 2i from the EB suspension culture 24 hours prior to stimulation with Activin A and BMP4 in order to generate brachyury expression (fig. 2). In summary, our protocol for the induction of mesoderm is as follows: 1) dissociation of adherent mES cell colonies with TrypLE, 2) resuspension at 1×10^5^ cells/ml in SFDM without LIF in petri dishes with 0.75/0.1 μM CHIR99021/PD0325901, 3) remove 2i after 72 (±3) hours by washing with PBS and resuspending in SFDM, and 4) stimulate with Activin A and BMP4 24 hours after removal of 2i.

### The Titration of Activin A and BMP4 Levels is Important for Successful Cardiomyocyte Differentiation

The relative concentrations of Activin A and BMP4 are important for directing differentiation to the mesodermal lineage and subsequently to cardiac mesoderm [4]. At this time Activin A and BMP4 are supplied by weight of cytokine and not standardized by an activity assay. As a consequence, different preparations of Activin A and BMP4 may have different amounts of “effective” cytokine and mass-based administration can lead to wildly varying differentiation results over time. Since the ratio of activities is critical for the induction of the lineage of interest, in this case cardiac mesoderm, it is imperative to test each lot for the optimal concentration of each cytokine to promote the desired differentiation. Nkx2–5 is a transcription factor expressed at the cardiac mesoderm stage of differentiation [20], [21]. We used mES cells expressing GFP driven by the Nkx2–5 promoter [15] to optimize the concentrations of five different lots of Activin A and five different lots of BMP4 used for cardiac differentiation. Table 2 shows a representative dose-response experiment where we varied the concentrations of BMP4, in the presence of 4 ng/ml of Activin A and 5 ng/ml VEGF, to determine the highest yield of Nkx2–5 positive cells after 28 hours of stimulation. This approach allowed us to determine the optimal concentration for each lot of cytokine for the formation of cardiac mesoderm, which is critical to maintain high yields of cardiomyocytes.

**Table 2:** Titration of Activin A and BMP4 improves cardiomyocyte yield.

**VEGF**	**5 ng/ml**				
**Activin A**	**0 ng/ml**	**4 ng/ml**			
**BMP 4**	**0 ng/ml**	**2.5 ng/ml**	**5 ng/ml**	**10 ng/ml**	**20 ng/ml**
**Day 5 Nkx2.5%**	0.52	6.6	12.7	8.9	11.9
**Day 8 cTroponin%**	2.28	37.6	53.3	48.3	25.5
**Day 8 Nkx2.5%**	0.3	27.4	34.6	28.5	11.2

EBs were formed from E14.Nkx2–5-GFP mES cells as above. On day 4 EBs were stimulated with Activin A 4 ng/ml and a range of BMP4 concentrations or unstimulated (0 ng/ml of both Activin A and BMP4). 28 hours (day 5+4 hours) after induction of cardiac mesoderm, EBs were dissociated with TrypLE. A subset of cells was analyzed for the expression of Nkx2.5 (GFP) using flow cytometry. The remaining cells were plated for continued differentiation into cardiomyocytes. On day 8 cells were stained for cardiac troponin (cTroponin) and analyzed for the percentage of cells expressing Nkx2.5 (GFP) and cTroponin using flow cytometry. Data are from a representative experiment that was performed at least 10 times utilizing 5 different lots of Activin A and 5 different lots of BMP4 for titration.

### Tracking Nkx2–5 Assists in Differentiation Optimization and Both Nkx2–5 and PDGFRα Expression Predict Cardiomyocyte Yield

**Figure 3:**
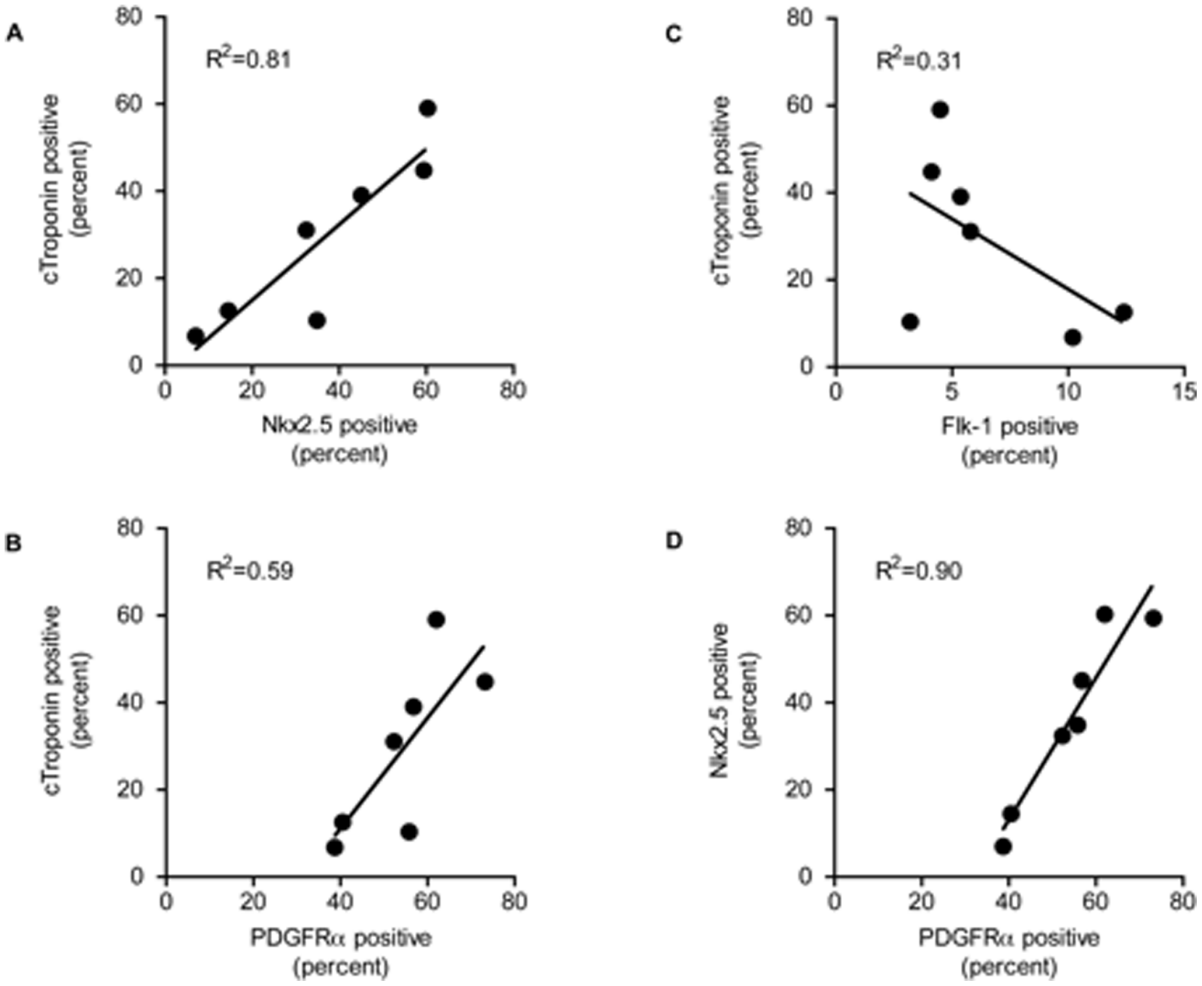
Tracking Nkx2–5 assists in differentiation optimization and both Nkx2–5 and PDGFRα expression predict cardiomyocyte yield. EBs were formed from E14.Nkx2-5-GFP mES cells in the presence of 0.75 μM CHIR99021 and 0.1 μM PD0325901. 72 (±3) hours after placing in suspension culture, the cells were collected, washed once to remove 2i, and resuspended in SFDM. 24 hours later (day 4) EBs were stimulated with Activin A 4 ng/ml, BMP4 10 ng/ml and VEGF 5 ng/ml. 28 hours (day 5+4hours) after induction of cardiac mesoderm, EBs were dissociated with TrypLE. A subset of cells was stained for PDGFRα and Flk-1 with fluorescent antibodies and the expression of Nkx2.5 (GFP), PDGFRα, and Flk-1 were assessed using flow cytometry. The remainder of the cells were plated for continued differentiation into cardiomyocytes. On days 7 to 12 the cells were dissociated and stained with an antibody to cardiac troponin (cTroponin) or the corresponding isotype control and the percentage of cardiac troponin expressing cells was assessed using flow cytometry. A) Comparison of the percentage of cTroponin expressing cells to the percentage of Nkx2.5 positive cells on day 5. B) Comparison of the percentage of cTroponin expressing cells to the percentage of PDGFRα positive cells on day 5. C) Comparison of the percentage of cTroponin expressing cells to the percentage of Flk-1 positive cells on day 5. D) Comparison of the percentage of PDGFRα positive cells on day 5 to the percentage of Nkx2.5 positive cells on day 5. Least squares fit simple linear regression was performed using data from 7 experiments and the R^2^ coefficient is reported in each panel.

Surface expression of Flk-1 and PDGFRα has been shown by Kattman et al. to assist in the assessment of efficacy of cardiac differentiation [3], [4]. We used linear regression to assess the correlation between surface marker expression and Nkx2–5 positivity early in cardiomyocyte differentiation (day 5) as well as correlation with future production of cardiomyocytes as judged by troponin positivity on day 7 or later. We found that PDGFRα expression reliably and reproducibly correlated with the percentage of Nkx2–5 positive cells across multiple experiments (fig. 3D). Furthermore, both Nkx2–5 and PDGFRα expression correlated with future development of cardiac troponin positive cells in multiple experiments (fig. 3A–B), explaining approximately 80% and 60% of the variability respectively. However, Flk-1 expression 28 hours after Activin A and BMP4 stimulation showed a negative correlation with the yield of cardiac troponin positive cells (fig. 3C). Since Nkx2–5 and PDGFRα individually correlate with later cardiac troponin expression in repeated experiments, each can be used in the assessment of differentiation efficiency at the cardiac mesoderm stage and in subsequent cardiac progenitors to predict later outcome.

### Low Serum Conditions Promote Cardiac Differentiation While Wnt Inhibitors are Dispensable

**Figure 4:**
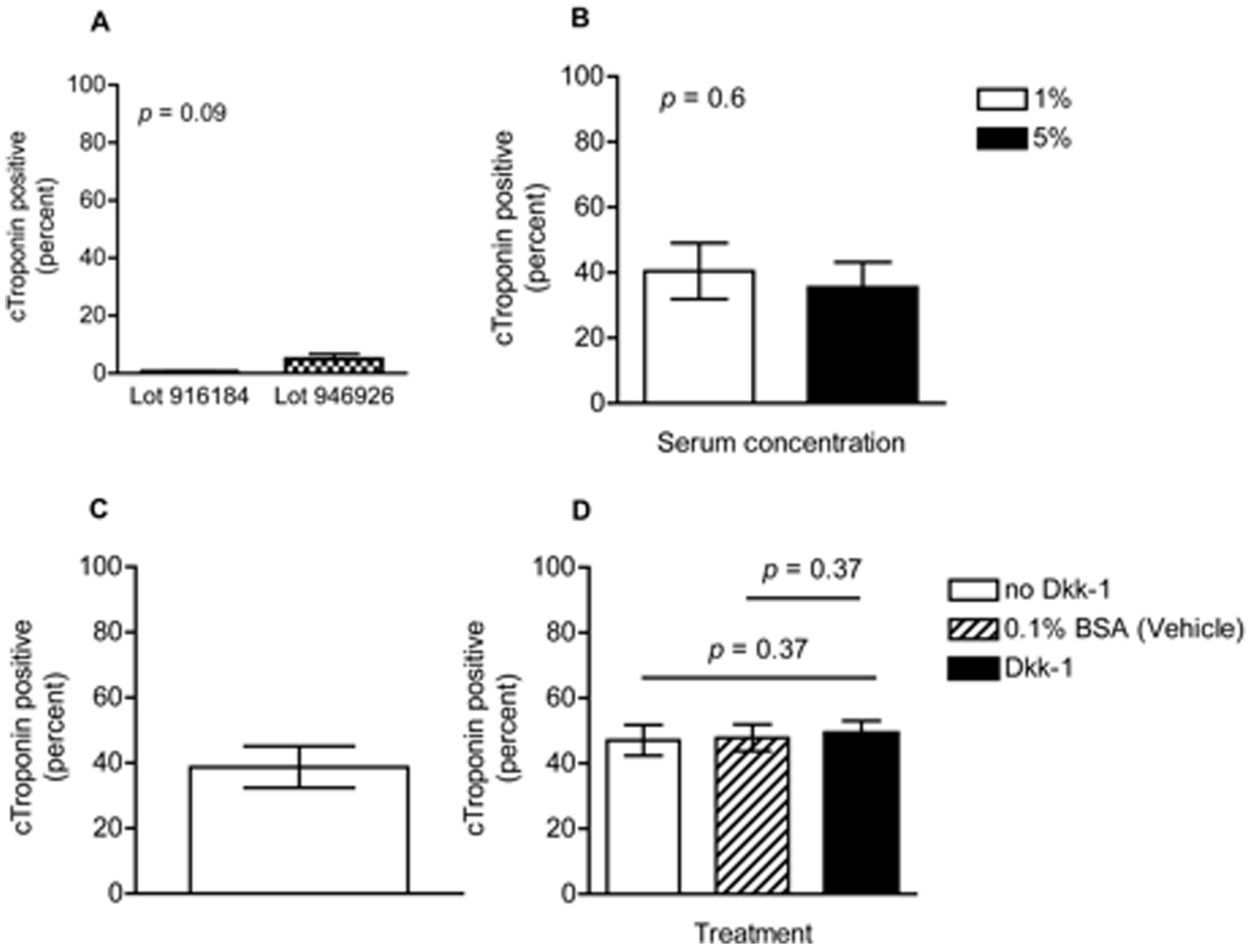
Low serum promotes cardiac differentiation while Wnt inhibitors are dispensable. E14.Nkx2-5-GFP mES cells were differentiated into cardiomyocytes as described. A) On day 5, cells were dissociated and plated in StemPro media with different lot numbers of StemPro Nutrient Supplement per the manufacturer’s instructions. The percentage of cardiac troponin (cTroponin) expressing cells was evaluated on days 10–12 as shown grouped by lot number of nutrient supplement. The average of 3 experiments ± SEM is reported. B) On day 5, cells were dissociated and plated in StemPro media with either 1% or 5% FBS. The percentage of cardiac troponin (cTroponin) expressing cells was evaluated on day 7. The average of 4 experiments ± SEM is reported. C) The average yield of cTroponin expressing cells ± SEM is shown for 12 experiments where the cells were cultured in StemPro with 1% FBS from day 5. D) On day 5, cells were dissociated and plated in StemPro media containing 1% FBS with or without the Wnt inhibitor Dkk-1 at 150 ng/ml or the corresponding vehicle control, 0.1% BSA. The percentage of cTroponin expressing cells was evaluated on day 7. The average of 3 experiments ± SEM is reported.

Many groups use StemPro differentiation media with the addition of the StemPro Nutrient Supplement for the culture of differentiating stem cells, however depending on the preparation of the nutrient supplement there is a large variability in the yield of cardiomyocytes (unpublished observations). Furthermore when we were able to obtain contracting cells using our differentiation protocol with StemPro Nutrient Supplement we only produced on average ∼3% cardiomyocytes (fig. 4A). We sought to determine if cardiac mesodermal cells could be supported in cell culture for continued differentiation to cardiomyocytes using fetal calf serum instead of the supplied nutrient supplement. We dissociated EBs following a 28 hour stimulation with Activin A, BMP4, and VEGF, then transferred these cells to adherent culture plates in StemPro media with 1%, and 5% FCS at a concentration of 1×10^6^ cells/ml at 300 μl per well in 96well plates (fig. 4B). We determined that a low level of serum, 1%, was sufficient to support the continued growth and differentiation of these cells to cardiomyocytes, and that this effect was reproducible with multiple lots of FCS. Using the protocol defined up to this time our average yield of cardiomyocytes was approximately 40% (fig. 4C). In addition, Wnt inhibition in the later stages of differentiation reportedly enhances cardiomyogenesis from mouse [22] and human ES cells [23]. We therefore tested the effect of Wnt inhibition using Dkk-1 on the yield of cardiomyocyte formation. As shown in figure 4D, inhibition of Wnt at the cardiac mesoderm stage did not improve the yield of cardiomyocytes. Lastly we investigated if there was a benefit to using FGF10 and bFGF following the formation of cardiac mesoderm as has been used by Kattman et al. [4]. We found that neither FGF10 nor bFGF were necessary to promote the formation of cardiomyocytes when added at the cardiac mesoderm stage (data not shown). Since we did not find a benefit from adding these factors, we removed them from our differentiation protocol in order to reduce complexity and avoid potential problems associated with the presence of additional factors.

### Lentiviral Infection of Mesodermal Precursors is Feasible to Induce Overexpression or Knockdown of Target Genes Prior to Cardiac Differentiation

**Figure 5:**
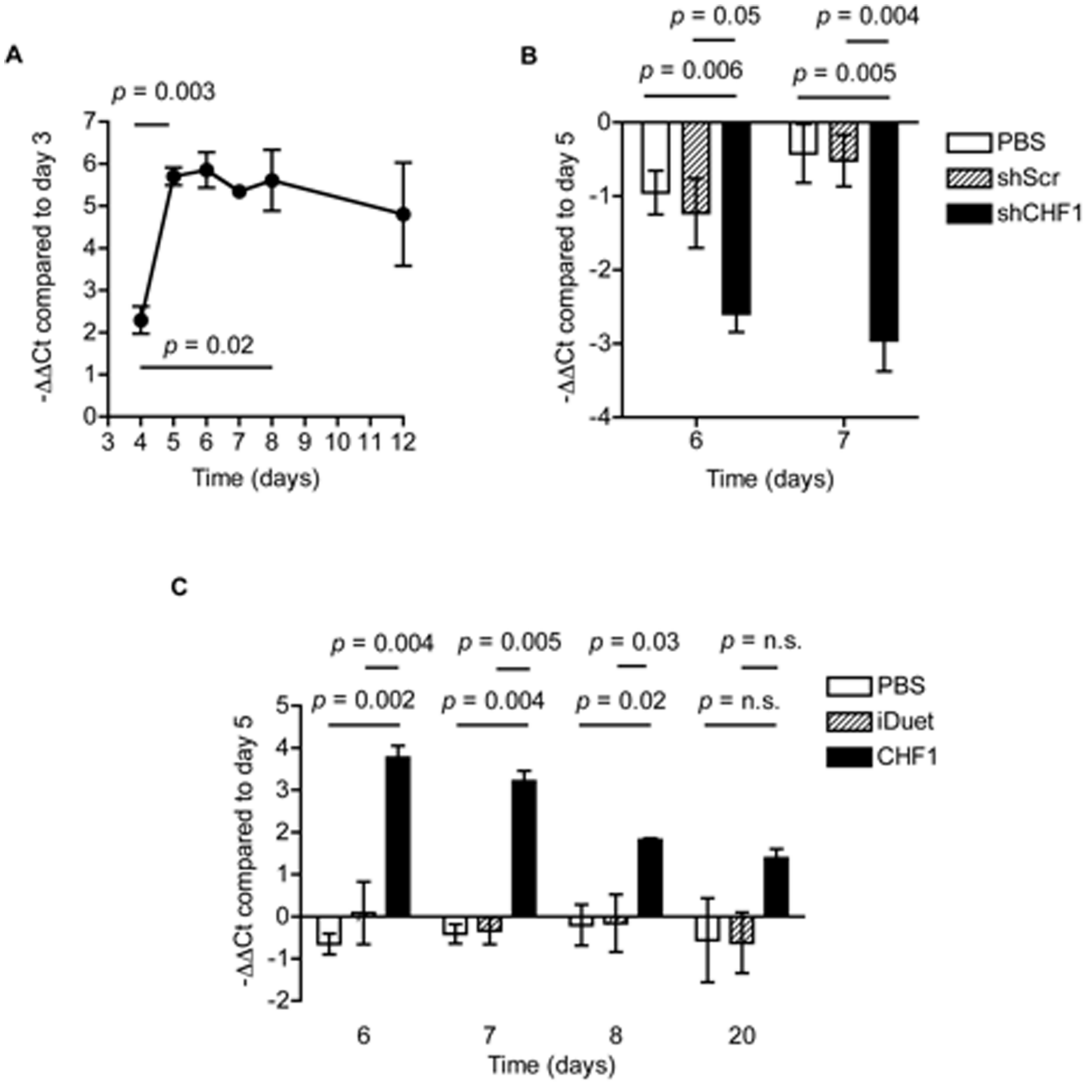
Lentiviral infection of mesodermal precursors is feasible to induce overexpression or knockdown of target genes. E14.Nkx2-5-GFP mES cells were differentiated into cardiomyocytes as described. A) On days 3 through 8 and 12 cells were lysed and RNA was collected. CHF1/Hey2 expression was assessed using qRT-PCR compared to mS16 as a control gene. The −ΔΔ Ct compared to day 3 for CHF1/Hey2 qRT-PCR is reported for each time point. The average of 3 experiments ± SEM is shown. B) On day 5 cells were treated with PBS, lentivirus containing scramble shRNA (shScr), or lentivirus containing CHF1/Hey2 shRNA (shCHF1); MOI 10. RNA was collected from cell lysates on day 5 prior to lentiviral transduction and on days 6 and 7. CHF1/Hey2 expression was assessed as above and the −ΔΔ Ct compared to day 5 is reported. The average of 4 experiments ± SEM is shown. C) On day 5 cells were treated with PBS, lentivirus containing empty iDuet vector (iDuet), or lentivirus containing the iDuet vector with CHF1/Hey2 constitutive overexpression (CHF1); MOI 10. RNA was collected from cell lysates on day 5 prior to lentiviral transduction and on days 6, 7, 8, and 20. CHF1/Hey2 expression was assessed as above and the −ΔΔ Ct compared to day 5 is reported. The average of 4 experiments ± SEM is shown.

In order to test modifiers of differentiation we must be able to manipulate the levels or activity of genes of interest at developmentally appropriate time points. In our protocol above, day 5 corresponds to the formation of cardiac mesoderm and a time in the protocol when cells are transitioned from large three dimensional EBs to individual cells. This is an opportune moment to introduce factors for the overexpression or the knockdown of genes of interest via viral transduction to assess their role in the differentiation of cardiac mesoderm into cardiac progenitors. In addition, cells from the same experiment can be infected with control viruses, which would remove confounding effects of interexperiment variability in differentiation efficiency. Accordingly, we used lentiviral transduction on day 5 to overexpress CHF1/Hey2 using a constitutively active construct as well as knockdown of CHF1/Hey2 using shRNA technology. Figure 5A shows the average endogenous CHF1/Hey2 expression over time through day 12 of our differentiation protocol. Figures 5B and C demonstrate the knockdown (8 fold on day 7) and overexpression of CHF1/Hey2 (8 fold on day 7, persisting to 3 fold on day 20) respectively following lentiviral transduction on day 5. These data demonstrate that lentiviral transduction on day 5 of our protocol is a feasible and effective method to overexpress and knockdown genes of interest to assess effects on cardiac differentiation.

### Knockdown of CHF1/Hey2 (day 5) Leads to Increased Expression of ANF and Cx43, but Does not Cause a Shift in Expression of Myosin Light Chain Isoforms

**Figure 6:**
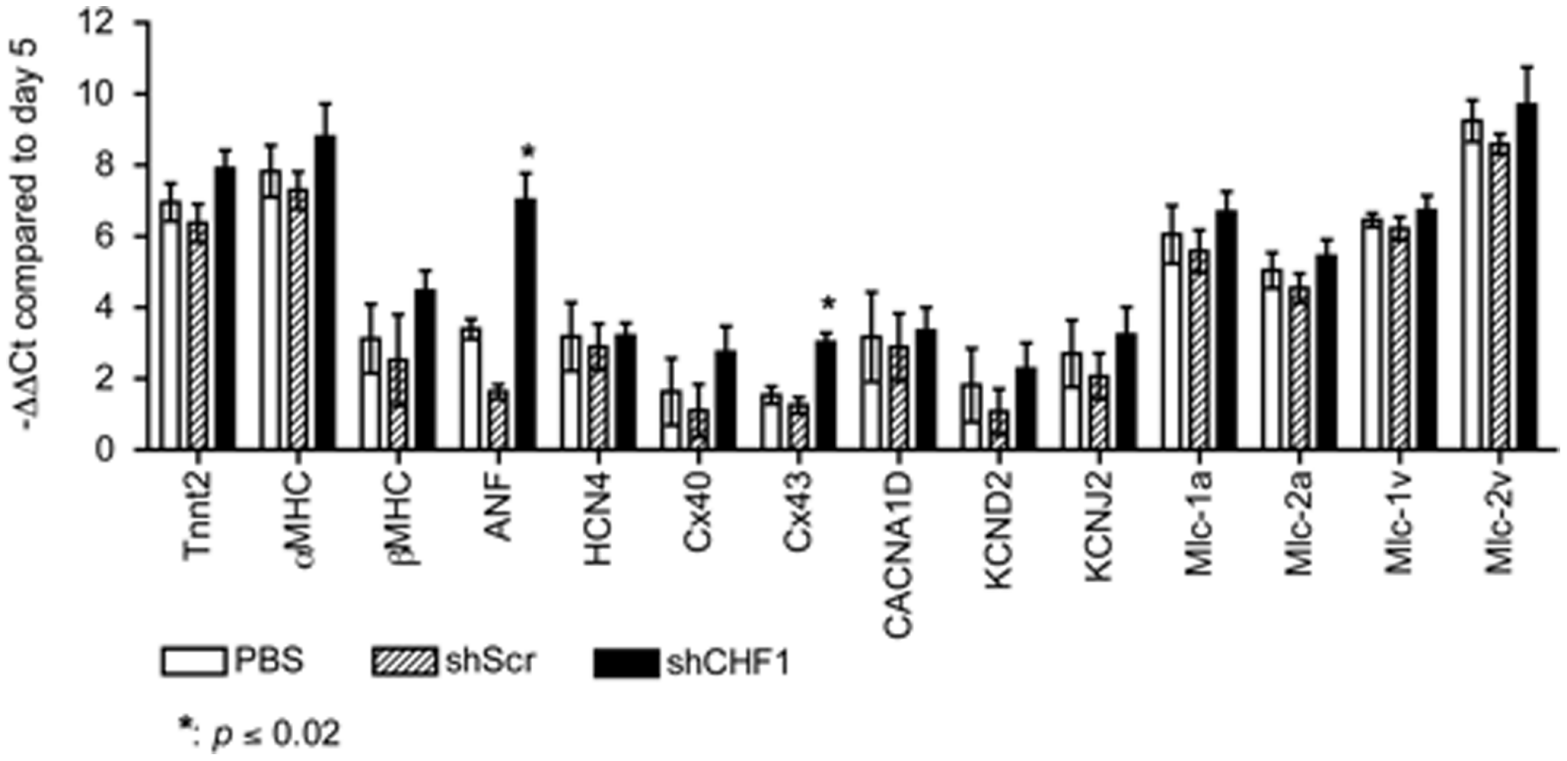
Knockdown of CHF1/Hey2 (day 5) leads to increased expression of ANF and Cx43. E14.Nkx2-5-GFP mES cells were differentiated into cardiomyocytes as described. On day 5 cells were treated with PBS, lentivirus containing scramble shRNA (shScr), or lentivirus containing CHF1/Hey2 shRNA (shCHF1); MOI 10. RNA was collected from cell lysates on day 5 prior to lentiviral transduction and on day 20. qRT-PCR was performed on 14 cardiac genes selected to interrogate cardiomyocyte subtypes, see table 1 for gene abbreviations used. The −ΔΔ Ct compared to day 5 is reported. The average of 3 experiments ± SEM is shown, * indicates p≤0.02 compared to both PBS and shScr controls.

CHF1/Hey2 loss of function has previously been associated with ectopic expression of atrial genes including atrial natriuretic factor (ANF), connexin 40 (Cx40), myosin light chain 1a (mlc-1a), and myosin light chain 2a (mlc-2a) in the developing mouse heart, thereby altering myocardial differentiation [24]. We hypothesized that the absence of CHF1/Hey2 would result in an increased number of atrial cardiomyocytes compared to ventricular cardiomyocytes after embryonic stem cell differentiation. To examine the effects of reduced CHF1/Hey2 on the differentiation of mES cells into cardiomyocyte subtypes, we generated a list of 14 genes that show preferential expression in either atrial, ventricular, nodal, or conduction system cardiomyocytes in developing embryos during late gestation (Table 1). We differentiated mES cells into cardiomyocytes based on the protocol described above and then used lentiviral transduction containing scramble shRNA or shRNA specific for CHF1/Hey2 as described above to knockdown CHF1/Hey2. We cultured the cells for a total of 20 days and then analyzed the RNA expression profile based on the 14 genes in Table 1. Figure 6 demonstrates that a reduction of CHF1/Hey2 early in the differentiation of mES cells into cardiomyocytes results in an increased expression of ANF by over 10-fold and connexin 43 (Cx43) by over 2.5-fold. However we did not observe any change in the expression profile of maturation markers such as αMHC and βMHC nor was there a change in the expression of the atrial and ventricular myosin light chain isoforms. These data demonstrate that there is a modest change in the profile of cardiomyocytes differentiated in the presence of reduced CHF1/Hey2, which reflects an alteration in the differentiation phenotype of the cells.

### Overexpression of CHF1/Hey2 (day 5) has No Effect on Cardiac Differentiation

**Figure 7:**
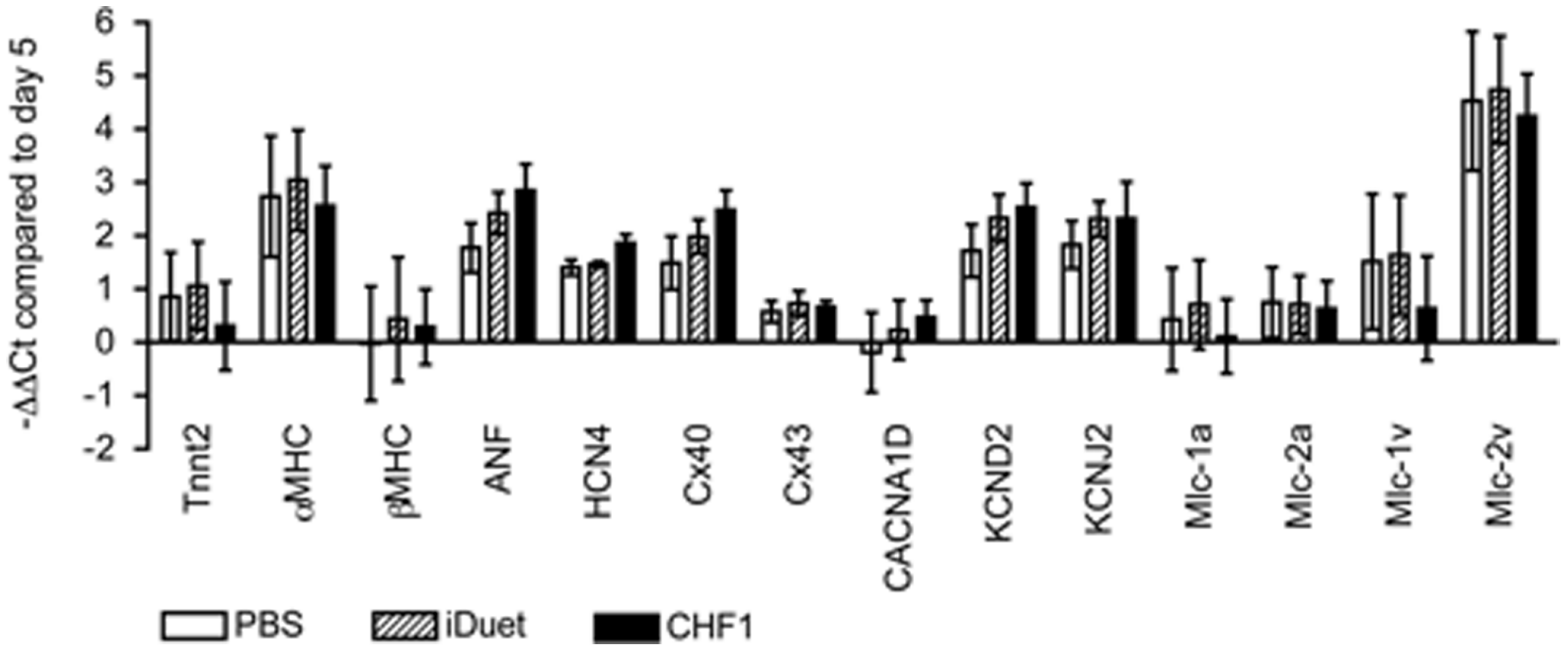
Overexpression of CHF1/Hey2 (day 5) has no effect on cardiac differentiation. E14.Nkx2-5-GFP mES cells were differentiated into cardiomyocytes as described. On day 5 cells were treated with PBS, lentivirus containing empty iDuet vector (iDuet), or lentivirus containing the iDuet vector with CHF1/Hey2 constitutive overexpression (CHF1); MOI 10. RNA was collected from cell lysates on day 5 prior to lentiviral transduction and on day 20. qRT-PCR was performed on 14 cardiac genes selected to interrogate cardiomyocyte subtypes, see table 1 for gene abbreviations used. The −ΔΔ Ct compared to day 5 is reported. The average of 3 experiments ± SEM is shown.

As a corollary, we hypothesized that overexpression of CHF1/Hey2 in the early stages of differentiation would result in fewer atrial and more ventricular cardiomyocytes. We again differentiated mES cells as described above then used lentiviral transduction to constitutively overexpress CHF1/Hey2 with PBS as a vehicle control and the empty iDuet vector as a lentiviral transduction control. We collected cells at day 20 and analyzed the RNA expression profile of the 14 genes used above. Although we were able to increase the expression of CHF1/Hey2 by 4-fold to 20-fold on differentiation days 6 to 8 (fig. 5C), which corresponds to the transition from cardiac mesoderm to cardiomyocytes in our protocol, we did not observe a change in the RNA profile of the cardiomyocytes at day 20 based on the 14 genes selected (fig. 7).

## Discussion

Although multiple protocols for the generation of cardiomyocytes from mouse ES cells exist, they vary in their technical complexity, variability and efficiency. Here we describe a simplified protocol for the generation of cardiomyocytes that is well-suited for assessing the effects of exogenous factors on cardiomyocyte differentiation from ES cells. The commonly used EB-based protocol with serum stimulation [1] generally yields <3% to 5% of total cells as cardiomyocytes [2]. This system is useful to test factors for an enhancement of cardiomyocyte differentiation, however, because of this low yield, detecting a reduction in differentiation efficiency would be challenging. Likewise a protocol with very high efficiency would not allow one to easily detect factors that enhance cardiomyocyte differentiation. Our protocol produces a yield of cardiomyocytes in the intermediate range of efficiency (30–40%) that allows for detecting either an increase or decrease in cardiomyocyte yield.

The 2i inhibitor cocktail with LIF maintains pluripotency in the absence of mouse embryonic fibroblasts [7] and 2i promotes embryoid body viability, but precise stepwise removal is essential to facilitate differentiation. The mechanisms by which they work have been postulated to involve the inhibition of ERK signaling (PD0325901) and inhibition of GSK3 (CHIR99021) [7]. The use of CHIR99021 is thought to maintain growth of pluripotent cells by preventing inhibition of biosynthetic pathways by GSK3. GSK3 inhibition is also predicted to promote canonical Wnt signaling by preventing the degradation of beta catenin [25] and to inhibit noncanonical Wnt signaling by preventing GSK3 mediated activation of Ror2 [26]. Wnt signaling is required for the formation of mesoderm from embryonic stem cells; however Wnt impairs the subsequent formation of pre-cardiac mesoderm and cardiac progenitors [27]. Noncanonical Wnt signaling has been implicated in cardiac differentiation [28]. Our data shows that the stepwise reduction and removal of this inhibitor is essential for efficient mesoderm formation, thereby suggesting that the effects are most likely mediated by its effects on activation of biosynthetic pathways and perhaps by inhibition of noncanonical Wnt signaling rather than by activation of canonical Wnt signaling, which would be expected to have the opposite effect.

Care and attention to detail are necessary to ensure the success of this protocol. In developing this protocol we discovered many pitfalls along the way. From the initial stages throughout the majority of the protocol we used TrypLE, trypsin like enzyme, in order to dissociate cells from adherent culture and from EBs. Although we did not systematically analyze the relative effects of trypsin and TrypLE, our impression was that TrypLE improved the yield of surviving cells at each step and reduced the impact of dissociation on cell passage. Here we demonstrate, as was previously shown by Kattman et al. [4], that the amounts of Activin A and BMP4 used must be evaluated with each different preparation of these factors. These factors are not supplied with a known relative activity per milligram and accordingly we recommend evaluation of each lot. EB size is also a factor in the efficient differentiation of cardiomyocytes [29]. As such there is an optimal time to stimulate with differentiation factors in order to produce cardiomyocytes. We found that approximately 4 days after EB formation and 24 hours after removal of 2i was ideal for stimulating with Activin A and BMP4. In addition, once EBs were formed, we did not disperse the EBs into individual cells before this stimulation. It is important to remember that this timing may differ depending on cell growth and rate of doubling and as such should be kept in mind when using this directed differentiation method. The stringency of the timing of each stage of the protocol is outlined in the Methods section and deviation from this timing produced inferior cardiomyocyte yields. Other factors such as length of time in cell culture, passage number, number of dissociation events during differentiation may also affect efficiency of differentiation, but these were not studied systematically.

To assist in optimizing our protocol, we used mES cells expressing brachyury-GFP and Nkx2-5-GFP to determine the transition to mesoderm and cardiac mesoderm respectively. These mES cell lines are useful in quality control of each step of the differentiation protocol and in troubleshooting timing issues should the yield of cardiomyocytes not be as expected. Kattman et al. demonstrated that one can monitor the development of cardiac mesoderm and cardiac progenitor cells using the surface proteins Flk-1 and PDGFRα [4]. The expression of Flk-1 is transient in cardiac progenitors, but is sustained in populations that become endothelium [3], [30]–[32]. Therefore, timing is an important factor in monitoring the quality of the differentiation protocol with Flk-1 expression. Here we observed that the expression of PDGFRα and Nkx2–5, but not Flk-1, 28 hours after stimulation with Activin A and BMP4 is predictive of the yield of cardiac troponin positive cells. This finding allows one to monitor the development of cardiac mesoderm/cardiac progenitors by simple analysis of PDGFRα surface expression in mES cells that are genetically unaltered and are not haploinsufficient in Nkx2–5 to produce cardiomyocytes.

The availability of a reliable protocol allows the investigation of factors that may affect cardiomyocyte differentiation as well as maturation and specification. A notable feature of our protocol is that we can effectively manipulate gene expression in the cardiac mesoderm stage using lentiviral transduction in part because we transduce the differentiating cells at a step when the cells are dissociated and each is exposed to the surrounding environment. Furthermore we are able to reduce the impact of inter-experiment variability on interpreting the effect of differentiation factors by using lentiviral transduction at the cardiac mesoderm stage in each differentiation experiment rather than using stable cell lines over- or under-expressing the gene of interest. This is because altering gene expression at the cardiac mesoderm stage effectively controls for the initial stages of differentiation from mES cell to cardiac mesoderm, which can differ from experiment to experiment. Overall this makes this system well-suited for evaluating factors which affect cardiomyocyte differentiation as well as cardiomyocyte subtype specification and maturation.

We hypothesized that CHF1/Hey2 would suppress the formation of atrial cardiomyocytes while promoting the development of ventricular cardiomyocytes. We used our system detailed here to test this hypothesis with shRNA based knockdown of CHF1/Hey2 and constitutive overexpression of CHF1/Hey2 timed to take effect when CHF1/Hey2 expression begins during differentiation. Based on previous animal data [24], we expected to see an increase in atrial genes such as ANF, Cx40, mlc-1a, and mlc-2a when CHF1/Hey2 was reduced. With CHF1/Hey2 expression reduced to 10 to 25% of native levels using shRNA we observed an increase in ANF and Cx43 expression without a difference in the other genes examined. ANF is expressed in the developing and mature atria, the developing trabecular ventricular myocardium, and the bundle branches, however it is restricted from nodal tissue and the compact ventricular myocardium [33], [34]. Cx43 is associated with high rates of conduction within the myocardium and is preferentially utilized by the ventricles compared to the atria [35], [36]. Cx43 is also expressed in the AV conduction system along with Cx40 [37]. Recently, it has been reported that *Hey2* common variants are associated with Brugada Syndrome [38], a condition characterized by abnormal atrial and ventricular conduction resulting in arrhythmogenesis. Notably we did not observe a change in the ratio of atrial and ventricular myosin light chain genes expressed. Taken together these findings suggest that reduced CHF1/Hey2 in early cardiac differentiation may be permissive for a conduction system phenotype to develop. Since Cx43 is expressed in a variety of murine cell types [39], it remains possible that the increase in Cx43 is coming from the non-cardiomyocyte population. However with similar levels of cardiac Troponin between treatments, the most likely explanation is that the increase in Cx43 is coming from the cardiomyocyte population. Since CHF1/Hey2 is expressed in developing cardiomyocytes and vascular cells, we cannot exclude the possibility that some of these gene expression changes may be occurring in a population of vascular cells. In contrast, we did not observe an alteration in the expression of the 14 genes examined with constitutive overexpression of CHF1/Hey2. Possible explanations include: 1) the majority of the cardiomyocytes already expressed CHF1/Hey2 and expression in the remaining minority did not produce a measurable effect, 2) the timing of overexpression was inadequate to alter the phenotype of the differentiating cardiomyocytes, 3) CHF1/Hey2 overexpression was limited to the non-cardiomyocyte populations, and 4) CHF1/Hey2 overexpression had other effects which are not reflected in our 14 gene panel.

Our efforts to develop a simplified, well-defined and reliable protocol for generating cardiomyocytes from mouse embryonic stem cells are limited by several caveats. Although we used two different embryonic cell lines in our study, we did not exhaustively characterize the differentiation of the brachyury-GFP cell line, as this work has been done by others [3], [4]. We did not exhaustively quantify every manipulation, nor did we examine the effectiveness of our protocol for additional lines. Our intent was not to exhaustively demonstrate reproducibility, but rather to demonstrate feasibility and provide a framework for investigators who are initiating these types of experiments. All conditions must be further optimized for each new cell line tested. Another caveat is that we did not perform more extensive morphological and functional characterization of our ES cell-derived cardiomyocytes following alteration of CHF1/Hey2 expression. Although such studies would possibly be useful in assessing effects of CHF1/Hey2 gain of function and loss of function on ES cell-derived cardiomyocyte phenotype, our preliminary studies suggest that any further effect is likely to be mild, so we did not pursue them.

In summary we have developed a simplified and reliable protocol for generating cardiomyocytes from mES cells that is well-suited for testing the effects of exogenous factors on cardiomyocyte differentiation, maturation, or subtype specification. We have shown that the cells are amenable to lentiviral transduction during the early cardiac mesoderm stage. Lastly we tested the effect of altered CHF1/Hey2 expression on cardiomyocyte differentiation and demonstrate that reduced CHF1/Hey2 expression following the cardiac mesoderm stage may be permissive for the formation of ventricular conduction system tissue.
